# A theory-informed systematic review to understand physical activity among women in Gulf Cooperation Council countries

**DOI:** 10.1186/s12889-023-15725-5

**Published:** 2023-05-30

**Authors:** Lujain A. Osabi, Joris van de Klundert, Sultana A. Alhurishi, J. M. Cramm

**Affiliations:** 1 Erasmus School of Health Policy & Management, Erasmus University Rotterdam, Rotterdam, The Netherlands; 2 School of Business, Universidad Adolfo Ibanez, Santiago de Chile, Chile; 3 Community Health Sciences Department, College of Applied Medical Sciences, King Saud University, Riyadh, Saudi Arabia

**Keywords:** Physical activity, Health belief model, Systematic review, Women, Gulf Cooperation Council countries

## Abstract

**Background:**

This systematic review was conducted to identify health beliefs and modifying factors influencing physical (in) activity among adult women in Gulf Cooperation Council (GCC) countries (Bahrain, Kuwait, Oman, Qatar, Saudi Arabia, and the United Arab Emirates).

**Methods:**

A comprehensive search of the Medline (Ovid), EMBASE, Cochrane Central, Web of Science, and Google Scholar databases was conducted to identify relevant articles published between 2009 and 2019. The quality of included studies was assessed using the Mixed Methods Appraisal Tool. Data collection and analysis based on the health belief model were performed to systematically examine the relationships of health beliefs and modifying factors to physical activity.

**Results:**

The sample comprised 15 studies (Saudi Arabia, *n* = 6; Oman, *n* = 5; Qatar, *n* = 2; Kuwait,* n* = 2). Reported physical activity prevalences were low (nearly 0% to 50%) and depended on the location, subpopulation, and measurement instrument. Evidence for relationships of modifying factors and health beliefs to physical activity was scarce and sometimes inconclusive. Among modifying factors, middle age and employment were associated positively with physical activity; marital status, educational level, income, and body mass index were not associated. Regarding health beliefs, the only conclusive evidence reported was that a lack of time was not associated significantly with physical activity in a population of men and women. Women reported a lack of social support and lack of skills significantly more frequently than men; these factors may explain the gender difference in physical activity prevalence. Differences in the reporting of fear of injury and lack of willpower were not significant.

**Conclusions:**

Robust qualitative and quantitative research on the contributions of health beliefs and modifying factors to the low prevalence of physical activity among women in GCC countries is urgently needed. Current evidence indicates that unemployed women, women aged < 25 years, and elderly women are less likely to be physically active. Women in this population are more likely than men to believe that a lack social support and skills affects their physical activity. Many known factors and health beliefs appear to be unrelated to physical activity among adult women in GCC countries.

**Supplementary Information:**

The online version contains supplementary material available at 10.1186/s12889-023-15725-5.

## Background

Low physical activity (PA) is an important risk factor for morbidity and mortality globally, associated with diseases such as stroke, diabetes, and ischemic heart disease and with the three leading health risk factors: high blood pressure, high body mass index (BMI), and high fasting plasma glucose level [1, 2]. It is among the top 10 such risk factors among females in the countries of the Gulf Cooperation Council (GCC) [2]: Bahrain, Kuwait, Oman, Qatar, Saudi Arabia, and the United Arab Emirates.

Although the health benefits of regular exercise and a physically active lifestyle are well known, PA is low among citizens, and especially women, of the GCC countries [3]. Proposed barriers to these women’s engagement in PA include the lack of indoor facilities (e.g., fitness centers) and outdoor facilities where they feel free to do so [4], and a general lack of social and cultural support or even a negative attitude toward it [5]. The social and cultural contexts in GCC countries are inclined toward male dominance, causing women to develop low self-esteem and confidence levels [6] that may inhibit their overcoming of barriers to PA [7].

Some initial efforts have been made to provide evidence for and advance our understanding of the factors explaining low PA among women in GCC countries [8]. To date, however, these efforts have made limited use of shared theoretical frameworks, thereby limiting the robustness of the results [9]. This lack of evidence and understanding may impede the effectiveness of policy making and the development of interventions to promote PA and reduce the morbidity and mortality that it causes [10].

**Fig. 1:**
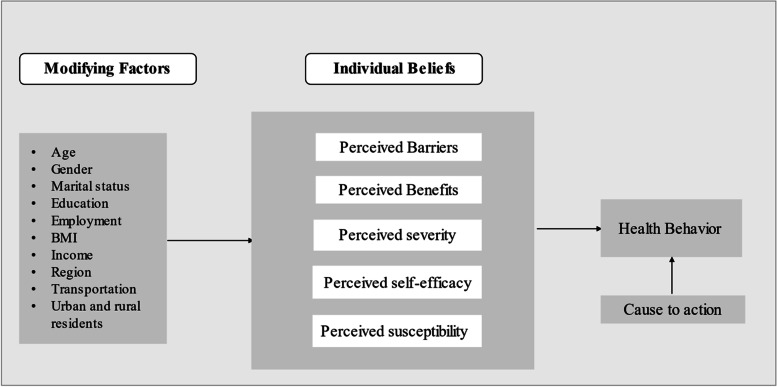
Health Belief Model Construct

The health belief model (HBM; Fig. 1) is a well-recognized theoretical framework designed to aid the understanding of individuals’ health behaviors, e.g., PA, and the factors explaining them [8]. It consists of six constructs – perceived benefits, barriers, susceptibility, and severity; self-efficacy; and cues to action – which, together with modifying factors such as age and gender, influence behavior [11–13]. A person’s decision and motivation to engage in a behavior depends on his/her awareness of the risk posed (perceived susceptibility), its seriousness (perceived severity), his/her confidence in its efficacy in reducing the risk of disease (perceived benefit), obstacles to taking action, and relevant other factors such as demographic characteristics (e.g., educational level, income). Cues to action act as catalysts and may fuel the desire to adopt specific health behaviors [13]. The HBM constructs and modifying sociodemographic factors together serve as intrinsic contextual variables explaining health behaviors of interest, such as PA. This model has been adopted widely and applied effectively to systematically review and synthesize evidence for a variety of health behaviors [14].

We conducted this systematic review to investigate PA among women in GCC countries using HBM constructs. The body of scientific literature on this topic is small and has not been systematically synthesized. The aim of this review was to improve the evidence base for and understanding of PA in this population, aiding the development of evidence-based health promotion policies and guiding future research.

## Methods

This systematic review was conducted according to the PRISMA guidelines [15], as reported in Supplement 1.

### Search strategy

A comprehensive search of the Medline (Ovid), EMBASE, Cochrane Central, Web of Science, and Google Scholar databases was conducted to identify relevant peer-reviewed publications dating to 2009–2019. The search terms were intentionally broad and are provided in Supplement 2.

### Study selection

Eligible studies 1) assessed the association between PA as a dependent variable and one or more factors that influence it as independent variables; 2) were conducted in one or more GCC countries; 3) had target populations of adults, including but not necessarily limited to women; and 4) were published in English. Studies 1) examining the prevalence of physical (in)activity only, without examination of corresponding factors; 2) conducted with populations with specific diseases (rather than general populations); 3) lacking explicit reporting on adults; and 4) examining PA as an independent variable were excluded. Gray literature was also excluded.

**Fig. 2:**
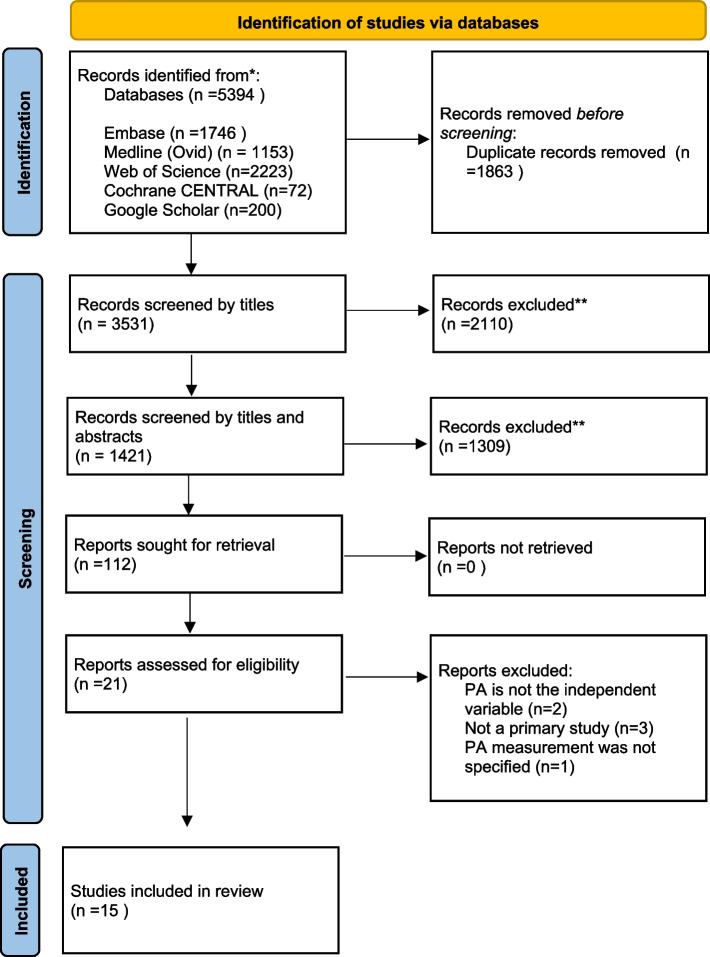
Search and selection process of the included studies

All identified articles were downloaded to Endnote reference management software (version X9; Clarivate Analytics). The search led to the identification of 2245 studies. Duplicates were removed, which reduced the sample to 1421 articles. Then, two researchers (LAO and SAA) independently assessed the titles and abstracts of the articles, applying the inclusion and exclusion criteria to determine eligibility. A total of 112 articles remained after this step. The reading of full texts led to the exclusion of 90 articles, after which 22 remained. In cases of disagreement or uncertainty (*n* = 7), the third and the fourth authors (JVK and JMC) were consulted. This led to a final sample of 15 articles (Fig. 2).

### Data extraction and analysis

Two authors (LAO and JVDK) extracted the following data from each publication into a Microsoft Excel spreadsheet: first author and year, study design, instrument (if any; e.g., a questionnaire or a step counter), PA, country, population, modifying factors, perceived susceptibility, perceived severity, perceived benefits, perceived barriers, self-efficacy, cues to action, and taking action. General, women-specific, and comparative (women vs. men) data were recorded. SAA independently verified the accuracy of data extraction.

The quality of the included studies was assessed using the Mixed Methods Appraisal Tool (MMAT) [16], with scores reflecting the number (0–5) of assessment criteria met. Studies with scores ≥ 4 were considered to be of high quality, and those with scores < 4 were considered to be of low quality (Supplements 2 and 3) [17].

## Results

**Fig. 3:**
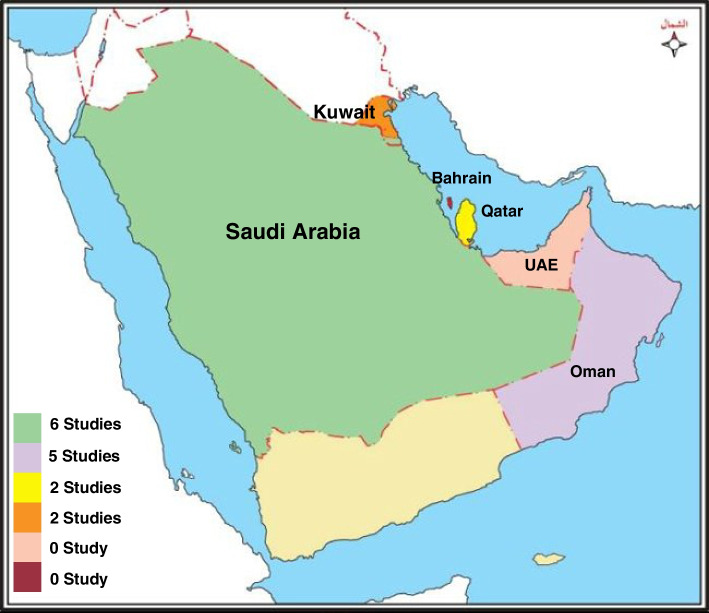
Geographical Distribution of the included studies

Six of the 15 included studies were conducted in Saudi Arabia [18–23] (one included Saudi and Egyptian college students), five studies were conducted in Oman [24–28], and two studies each were conducted in Qatar [29, 30] and Kuwait [31, 32] (Fig. 3). Some reports provided results for women only, whereas others provided results on women and men, separately or combined, and/or on women versus men. According to MMAT scores, all studies but one [32] were of high quality. The MMAT assessments are presented in Supplement 3 and the extracted data are provided in Supplement 4.

Data on the prevalence of PA are provided in Table 1. The reported prevalence of PA among women varied widely, ranging from 1.9% to 53%. This variety may be explained by the differences in contexts, study populations, and in the definitions of PA that varied across studies (see Supplement 4 for details). Six studies showed that PA levels were significantly lower among women than among men. Two studies, which included subpopulations of college students and primary care attendants, respectively, revealed no significant gender difference in PA. One report described a difference but did not indicate its significance. For the general population, the maximum reported PA prevalence was 38.2%.

**Table 1:** Included studies, main characteristics, and physical activity prevalence

No	Study	Country	Sample size	Sample size of female	Age groups	Location	% PA in Females	% PA in Males	% PA in all participants	Significant differences	Non-significant differences
1	Al-Nozha et al. (2007)[18]	Saudi Arabia	17,395	9098	30–39 40–49 50–59 60–70	Primary healthcare centers	1.9 (1.6–2.2)	6.1 (5.6–6.6)	-	Women were significantly less likely to be PA	-
2	AlQuaiz &Tayel (2009)[19]	Saudi Arabia	450	144	15–29 30–45 > 45	Primary Health care clinics	12.4	28.5	17.6	Women were significantly less likely to be PA	-
3	Abaozaid & Farahat (2010)[20]	Saudi Arabia	329	139	< 50 ≥ 50	Family medicine clinics in Hospitals	53	54	-	-	No significant differences in PA between female and male primary care patients
4	Al-Isa et at (2011)[31]	Kuwait	787	409	-	University	44.5	66.4	-	Female college students were significantly less likely to be PA	-
5	El-Gilany & El-Masry (2012)[21]	Saudi Arabia Egypt	616	213	< 20 ≥ 20	Universities	-	-	84.7 for Egyptian students, 58.9% for Saudi students	-	No significant difference in PA between female and male college students
6	Mabry et al. (2012)[24]	Oman	1335	744	20–29 30–39 ≥ 40	Household survey	-	overall prevalence of inactivity in men in Sur City: 31.2%, 23.8% for transport and 42.7% for leisure inactivity	overall prevalence of inactivity in men in Sur City: 31.2%, 23.8% for transport and 42.7% for leisure inactivity	Significantly higher odds of leisure inactivity were seen in men	-
7	Allafi & Waslien (2014)[32]	Kuwait	1370	-	average age was 26	Online Survey	-	-	-	-	-
8	Al-Zalabani et al. (2015)[22]	Saudi Arabia	4758	2418	15–24 25–34 35–44 45–54 55–64	Household survey	27.1	39.9	33.3	Women were significantly less likely to be PA	-
9	Al Sayegh et al. (2016)[29]	Qatar	549	549	18–24 25–44 45–64	Community based program	23.5	-	-	-	-
10	Mabry et al. (2016)[25]	Oman	2977	1486	18–29 30–39 ≥ 40	Household survey	41.6 (95% CI:38.0–45.2)	54.2 (95% CI:50.5–57.9)	-	Women were significantly less likely to be PA	-
11	Alghafri et al. (2017)[27]	Oman	312	175	≤ 57 > 57	Primary health care clinics	-	-	17	-	-
12	Alghafri et al. (2017)[28]	Oman	312	175	< 40 40–49 50–59 60–69 ≥ 70	Primary health care clinics	12	35	-	Women were significantly less likely to be PA	-
13	Mabry et al. (2017)[26]	Oman	2977	1487	18–39 ≥ 40	Household survey	38.2	48.0	-	-	-
14	Shabaan et al. (2018)[30]	Oatar	1454 (summer) 630 (winter)	-	< 20 20–50 ≥ 50	Popular neighborhood	-	-	-	Women are significantly more likely to practice outdoor walking in the weekend (instead of weekdays)	No significant differences in seasonal patterns for walking behavior between women and men
15	Almutairi et al. (2018)[23]	Saudi Arabia	1656	1165	< 20 21–30 ≥ 31	University	-	-	6–24%	males were more willing to engage in physical activity than females	-

“ -” indicates no data were reported

For brevity “PA” is used as an abbreviation for “Physically Active” and as an abbreviation for “Physical Activity”

As none of the included articles contained reporting on the effect of perceived susceptibility or severity on PA, we do not report on them. First, however, we report findings for modifying factors (Table 2). Thereafter, we consider these factors together with the beliefs (Table 3). In the tables, results for both sexes combined are provided for studies that did not provide separate results for women or that additionally provided combined results for both sexes. In addition, comparative data (i.e., women vs. men) are provided in Table 3; the articles contained no such data for the modifying factors. We report findings only for factors and beliefs considered in at least two studies.

**Table 2:** Relationships between Modifying Factors and PA

**Factor**	**Study**	**Results for Women**		**Results for both sexes together**	
		**Sig**	**Non-Sig**	**Sig**	**Non-Sig**
Age	Al-Nozha et al (2007)[18]	Women of Age ≥60 are significantly less likely to be PA than women of age 30-49.	No significant differences between age groups 30-39. 40-49, 50-59.	NIF	NIF
	AlQuaiz and Tayel (2009)[19]	NIF^a^	NIF	Respondents (patients) younger than 45 were significantly less likely to be PA	NIF
	AboZaid and Farahat (2010)[20]	NIF	NIF	Respondents of age 50 and older significantly less likely to be PA	NIF
	Al Isa et al. (2011)[31]	NIF	NIF	NIF	Age was not significant (among college students)
	El-Gilany and El-Masry (2012)[21]	NIF	NIF	NIF	Age was not significant (among college students)
	Mabry et al. (2012)[24]	Sitting time decreased significantly with age. For women who worked, PA was significantly lower for age ≥ 40	NIF	NIF	NIF
	Alaffi (2014)[32]	NIF	NIF	NIF	Older age is positively associated with sedentary behavior (significance unclear)
	Al-Zalabani (2015)[22]	Women in the 25-34 years, 35-44 years and 45-54 years age groups are more likely to be PA than women of age ≥ 55	Women of age < 25 were not less likely to be PA than women of age ≥ 55	The age group 35-44 was significantly less PA than the age group 55+	PA for age groups < 35 and 45-54 was not significantly different from 55+
	Mabry et al. (2016)[25]	PA significantly decreased with age.	NIF	NIF	NIF
	Sayegh et al.(2016)[29]	Women aged 18-24 were significantly less active than women of age 45-64.	Age group 25-44 was not significantly different from 18-24 or 45-64.	NIF	NIF
	Alghafri et al. (2017)[27] BMC	NIF	NIF	T2D patients of age ≤ 57 were significantly likely to be PA.	Age was not significantly associated with PA for categories <40, 40-49, 50-59,60-69, ≥70.
	Mabry et al. (2017)[26]	Younger women were significantly more likely to sit less than 7 hours per day than Women of age ≥ 40	NIF	NIF	NIF
	Almutairi et al (2018)[23]	NIF	For college students, age was not significantly associated with PA.	NIF	NIF
Marital status	Al-Nozha et al.(2007)[18]	NIF	NIF	Divorced/Widowed respondents were significantly less likely to be PA than married or single respondents	No significant differences between single and married respondents.
	AlQuaiz and Tayel (2009)[19]	NIF	NIF	NIF	No significant difference in PA prevalence between married and unmarried respondents.
	Abozaid, H. A. and F. M. Farahat (2010)[20].	NIF	NIF	NIF	No significant association was detected between marital status and physical activity
	Al-Isa et al. (2011)[31]	NIF	NIF	Married students were significantly less likely to be PA	NIF
	Mabry (2012)[24]	NIF	Marital status was not significantly related to PA.	NIF	NIF
	Alghafri et al. (2017)[27] BMC	NIF	NIF	NIF	Single T2D patients were more likely to be PA than married/divorced/widowed T2D patients.
	Mabry et al. (2017)[26]	Marrried women of age ≥ 40 were more likely to be PA	No significant differences for age < 40.	NIF	NIF
Education	Al-Nozha et al.(2007)[18]	NIF	NIF	Respondents with a College/University background were significantly more likely to be PA than respondents from all other categories (secondary, primary, read/write, illiterate). Respondents with secondary or primary education with significantly more likely to be PA than respondents without (read/write, illiterate).	No significant differences between respondents with secondary educations versus respondents with primary education only. No significant differences between illiterate respondents and respondents who have not completed primary education yet were able to read/write.
	AlQuaiz and Tayel (2009)[19]	NIF	NIF	NIF	No significant difference in PA prevalence between respondents with versus without university degree
	Abozaid et al. (2010)[20].	NIF	NIF	NIF	No significant association was detected between level of education (from illiterate to university) and PA
	Mabry et al.(2012)[24]	NIF	Education was not significantly associated with (various forms of) PA.	NIF	NIF
	Al-Zalabani (2015)[22]	NIF	No significant differences in PA between higher educated females, compared to lower or medium educated.	Medium Educated respondents were significantly more likely to be PA, compared to higher educated.	No significant differences between lower and higher educated respondents.
	Mabry et al (2016)[25]	NIF	Education (from illiterate to secondary plus) was not associated wih PA	NIF	NIF
	Alghafri et al. (2017)[27] BMC	NIF	NIF	T2D patients who completed a university degree were significantly more likely to be PA.	No significant differences among T2D patients with education ranging from college complete to illiterate.
	Mabry et al (2017)[26]	NIF	Education (from illiterate to secondary plus) was not associated wih PA	NIF	NIF
	Almutairi et al (2018)[23]	College students with more years in college were less likely to PA.	NIF	NIF	NIF
Employment	Abozaid et al (2010)[20]	NIF	NIF	Employed respondents were significantly more likely to be PA	NIF
	Mabry et al.(2012)[24]	Employment was significantly and positively associated with PA and sitting time	NIF	NIF	NIF
	Al-Zalabani (2015)[22]	Employed women were significantly more likely to be PA than retired/unemployed.	PA of Students and homemakers was not significantly different from retired/unemployed	Non-governmental employees were significantly more likely to be PA than retired/unemployed	PA of governmental employees and students was not significantly different from retired/unemployed.
	Mabry et al. (2016)[25]	Employed women were significantly more likely to be PA than unemployed, for age 18-29	No significant difference between employed and umemployed women of ages ≥ 30.	NIF	NIF
	Alghafri et al. (2017)[27] BMC	NIF	NIF	Governmental employees were significantly more PA than other employees, self-employed, unemployed, retired	No significant differences between non-governmental employees, self-employed, unemployed, retired
	Mabry et al. (2017)[26]	NIF	No significant differences in sitting time for employed versus unemployed women.	NIF	NIF
BMI	Mabry et al.(2012)[24]	NIF	BMI was significantly associated with PA.	NIF	NIF
	Al-Nozha et al.(2007)[18]	Average BMI was significantly lower for PA women.	BMI was not significantly associated with PA (no differences between BMI categories < 25, 25-30, >30).	NIF	NIF
	Abozaid et al. (2010)[20]	NIF	NIF	NIF	Obesity was not significantly associated with PA.
	Al-Isa et al. (2011)[31]	NIF	NIF	BMI ≤ 30 was significantly and positively associated with PA.	NIF
	Alaffi et al (2014)[32]	NIF	NIF	Unhealthy exercise behaviors increased significantly with BMI.	NIF
	Sayegh et al. (2016)[29]	NIF	No significant relationship between BMI and PA.	NIF	NIF
	Alghafri et al. (2017)[27] BMC	NIF	NIF	NIF	No significant relationship between BMI and PA.
Location	Al-Nozha et al.(2007)[18]	NIF	NIF	Respondents from the Southern region were significantly more PA than for all other regions. Respondents from the Western region were significantly more PA than respondents from Central, Eastern, and Northern region.	No significance differences between respondents from Central, Eastern and Northern regions.
	El Gilany et al (2011)[21]E	NIF	NIF	Egyptian college students were significantly more likely to be PA than Saudi counterparts.	NIF
	Al Zalabani et al. (2015) [22]	Women from the central, eastern and northern regions of Saudi Arabia are significantly less likely to be PA	NIF	Respondents from the Southern region were significantly more PA than respondents from the central and northern region of Saudi Arabia	PA of respondents from the Eastern and Western regions was not significantly different from respondents from the Southern region of Saudi Arabia.
	Mabry et al. (2016)[25]	Region was significantly associated with PA for women of age 30-39.	Region was not significantly associated with PA for women < 29 or ≥ 40.	Region was significantly associated with PA for regions of Oman	NIF
	Alghafri et al. (2017)[27] BMC	NIF	NIF	NIF	No significant relationship between region and PA.
Income	Alquaiz et al (2009)[19]	NIF	NIF	NIF	Income was not significantly associated with PA.
	Al Isa et al. (2011)[31]	NIF	NIF	NIF	Family income was not significantly associated with PA (among college students) (among college students)
	El-Gilany et al. (2012)[21]	NIF	NIF	NIF	Family income was not significantly associated with PA.
	Al Zalabani et al. (2015) [22]	NIF	Family income was not significantly correlated to PA	NIF	Family income was not significantly correlated to PA
	Mabry et al. (2016)[25]	NIF	Wealth quintile was not significantly associated with PA	NIF	Wealth quintile was not significantly associated with PA
Urban and rural residents	Al-Nozha et al.(2007)[18]	NIF	NIF	NIF	No significant difference in PA between urban and rural residents
	El Gilany et al. (2011) [21]	NIF	NIF	NIF	No significant difference in PA between college students of rural versus urban origin.
Health Status	Abozaid et al. (2010)[20]	NIF	NIF	Diabetes Patients were less likely were less likely to be PA.	Respondents suffering from hypertension, obesity, or practiced smoking were not significantly less likely to be PA.
	Al Isa et at (2011)[31]	NIF	NIF	NIF	No significancy between prevalence of chronic disease and PA.
	Alghafri et al. (2017)[27] BMC	NIF	NIF	T2D Patients suffering from comorbidities were less likely to be PA.	None of the T2D measures was significantly associated with PA.

^a^*NIF* No information found

**Table 3:** Health Belief Model constructs associated with PA

**HBM Component**		**Study**	**Women Only**		**All Respondents**		**Men vs. Women**	
			**Significant**	**Non-Significant**	**Significant**	**Non-Significant**	**Significant**	**Non-Significant**
Perceived Barriers	Lack of resources	AlQuaiz, A. M. and S. A. Tayel (2009)[19]	NIF^a^	Confirmed as a barrier by 41.8% of female respondents	Significantly and negatively associated with income	Not significantly associated with age, education, or marital status	Confirmed significantly more frequently by women	NIF
		Alghafri et al. (2017)[27]	NIF	Reported as a barrier by 29.1% of female respondents	Significantly and positively associated with being married. Significantly and negatively associated with income	Not significantly related with PA. Not significantly associated with age employment, or education	NIF	No significant difference
	Lack of access	El-Gilany, A. H. and R. El-Masry (2012)[21]	NIF	NIF	NIF	No significant differences in confirmation frequency of lack of access/safe sporting facilities between Saudi (67.7%, 56.9%) and Egyptian students (61.8%, 52.0%)	NIF	NIF
		Abozaid, H. A. and F. M. Farahat (2010)[20]	NIF	NIF	NIF	“No Place” was not significantly related to PA	NIF	NIF
	Lack of time	AlQuaiz, A. M. and S. A. Tayel (2009)[19]	NIF	Confirmed as a barrier by 82.2% of female respondents	NIF	Not significantly associated with age, education, marital status, or income	NIF	No significance difference
		El-Gilany, A. H. and R. El-Masry (2012)[21]	NIF	NIF	Saudi students (89.9%) experienced significantly more time constraints (and/or other priorities) than Egyptian students (84%)	NIF	NIF	NIF
		Abozaid, H. A. and F. M. Farahat (2010)[20]	NIF	NIF	NIF	No significantly related with PA	NIF	NIF
		Alghafri et al. (2017)[27]	NIF	Reported as a barrier by 15.4% of female respondents	Significantly and positively associated with age (≥ 57), being married, employed, education, and income	No significantly related with PA	Confirmed significantly less frequently by women	NIF
	Lack of social support	AlQuaiz, A. M. and S. A. Tayel (2009)[19]	NIF	Confirmed as a barrier by 82.6% of female respondents	NIF	Not significantly associated with age, education, marital status, or income	Confirmed significantly more frequently by women	NIF
		El-Gilany, A. H. and R. El-Masry (2012)[21]	NIF	NIF	Saudi students (24.6) reported significantly more frequently to lack a a person to care for the family while engaging in PA than Egyptian students (16%)	No significant differences in confirmation frequency of lack of friends/social support and encouragement/ objections by parents between Saudi (58.2%, 45.8%, 28,6%) and Egyptian students (57.7%, 53.6%, 25.7%)	NIF	NIF
		Alghafri et al. (2017)[27]BMJ Open	NIF	Reported as a barrier by 35.4% of female respondents	NIF	Not significantly related with PA. Not significantly associated with age (≥ 57), being married, employed, education, and income	Confirmed significantly more frequently by female respondents	NIF
	Fear of Injury	AlQuaiz, A. M. and S. A. Tayel (2009)[19]	NIF	Confirmed as a barrier by 22.9% of female respondents	Confirmed significantly more frequently by respondents with higher age, education below university level, married, and lower income	NIF	NIF	No significance difference
		El-Gilany, A. H. and R. El-Masry (2012)[21]	NIF	NIF	Saudi students (88.4%) experienced significantly more time constraints (and/or other priorities) than Egyptian students (24.4%)	NIF	NIF	NIF
		Alghafri et al. (2017)[27]	NIF	Reported as a barrier by 26.3% of female respondents	Significantly and negatively associated with PA. Significantly and negatively associated with age (≥ 57) employment, and education	Not significantly associated with being employed or married	NIF	No significance difference
	Environmental Barriers	El-Gilany, A. H. and R. El-Masry (2012)[21]	NIF	NIF	“Unsuitable Climate” was reported significantly more frequently by Saudi students (51.5%) than by Egyptian students (4.3%)	NIF	NIF	NIF
		Alghafri et al. (2017)[27]	NIF	Reported as a barrier by 13.1% of female respondents	NIF	Not significantly related with PA. Not significantly associated with age (≥ 57), being married employment, education, or income	NIF	NIF
Perceived Benefits	Weight Control/ Obesity Prevention	El-Gilany, A. H. and R. El-Masry (2012)[21]	NIF	NIF	NIF	No significant difference in reporting between Saudi (66.35%) and Egyptian students (64.6%)	NIF	NIF
		Abozaid, H. A. and F. M. Farahat (2010)[20]	NIF	NIF	NIF	No significant effect on PA	NIF	NIF
	Maintain health	Abozaid, H. A. and F. M. Farahat (2010)[20]	NIF	NIF	NIF	No significant effect on PA	NIF	NIF
		El-Gilany, A. H. and R. El-Masry (2012)[21]	NIF	NIF	NIF	No significant difference in reporting between Saudi and Egyptian students (72.4%)	NIF	NIF
	Fun and enjoyment	Abozaid, H. A. and F. M. Farahat (2010)[20]	NIF	NIF	NIF	No significant effect on PA	NIF	NIF
		El-Gilany, A. H. and R. El-Masry (2012)[21]	NIF	NIF	NIF	No significance difference in reporting between Saudi (52.9%) and Egyptian students (48.6%)	NIF	NIF
Self Efficacy	Lack of will- power	AlQuaiz, A. M. and S. A. Tayel (2009)[19]	NIF	Confirmed as a barrier by 78.9% of female respondents	NIF	Not significantly associated with age, education, marital status, or income	NIF	No significant difference
		Alghafri et al. (2017)[27]	NIF	Reported as a barrier by 48.6% of female respondents	Significantly and negatively associated with PA. Significantly and negatively associated with income	Not significantly related with PA. Not significantly associated with age (≥ 57), being married, or education	NIF	No significant difference
	Lack of skills	AlQuaiz, A. M. and S. A. Tayel (2009)[19]	NIF	Confirmed as a barrier by 44.0% of female respondents	Confirmed significantly more frequently by respondents with education below university level and y respondents with lower income	Not significantly associated with age or marital status	Confirmed significantly more frequently by women	NIF
		El-Gilany, A. H. and R. El-Masry (2012)[21]	NIF	NIF	NIF	Lack of sport skills/inability to practice sport was not different in reported frequency between Saudi (50,5%,,40.4%) and Egyptian (48.6%, 41.4%) students	NIF	NIF
		Alghafri et al. (2017)[27]	NIF	Reported as a barrier by 28.3% of female respondents	Significantly and negatively associated with age (≥ 57) employment, and education	Not significantly related with **PA**. Not significantly associated with being employed or married	Confirmed significantly more frequently by women	NIF
	Lack of energy/power	AlQuaiz, A. M. and S. A. Tayel (2009)[19]	NIF	Confirmed as a barrier by 77.0% of female respondents	NIF	Not significantly associated with age, education, marital status, or income	Confirmed significantly more frequently by women	NIF
		El-Gilany, A. H. and R. El-Masry (2012)[21]	NIF	NIF	NIF	Lack of or low physical power/ feeljng tired was not different in reported frequency between Saudi (35.4%) and Egyptian (30.1%) students	NIF	NIF
		Alghafri et al. (2017)[27]	NIF	Reported as a barrier by 16.0% of female respondents	Significantly and positively associated with being employed, and with being educated	Not significantly related with **PA**. Not significantly associated with age (≥ 57), being married, or income	NIF	No significant difference

^a^*NIF* No information found

### Modifying factors

The factors found to modify PA levels in the included studies were age, marital status, educational level, employment status, BMI, region, income level, health status, and residence (urban vs. rural).

The effect of age on PA was considered in 13 studies, with little uniformity in the age categories used. Results for women are presented in seven articles. Age was found to have no significant effect in one study conducted with female college students [23]. Four studies [18, 22, 24, 25] showed that women were less likely to engage in PA after a certain age (which ranged from 40 to 60 years), whereas two studies [26, 29] found no significant effect of older age categories on PA. The two studies that included women aged ≤ 25 years showed that these women were significantly less active [29] or formed the only group not significantly more physically active than women aged ≥ 55 years [22]. Findings for both sexes combined, reported in six articles, varied widely. In two studies conducted with college students, age was not associated significantly with PA [21, 31].

Of six articles providing data on the relationship of marital status to PA, two provided such data for women only [24, 26]. The only significant difference reported was that married women aged ≥ 40 years were significantly more physically active than their unmarried counterparts [26]. For general populations, the results were slightly mixed. Three of five studies reported no difference in PA related to marital status [18–20], with the exception that single respondents were more physically active than were divorced/widowed respondents [18, 27]. Similarly, married college students were reported to be significantly less physically active than their single peers [31].

Reports on five of the nine studies in which the relationship between the education level and PA was examined provided results for women only [22–26]. The only significant association found was that the PA level of female college students declined significantly with an increasing number of years in college [23]. Two of five reports that provided data for both sexes combined revealed no significant difference in PA related to the educational level [19, 20]. In two studies, individuals with college/university degrees were found to be significantly more likely to engage in PA than were less-educated individuals, with no difference in PA among the less-educated categories [18, 27]. In another study, medium education levels were associated with a greater likelihood of PA [22].

All three studies in which the relationship between women's employment status and PA was examined showed that this relationship was significant and positive. In one of these studies [25], this relationship was significant only for women aged 18–29 years. A fourth study revealed no significant relationship between employment status and sitting time [26]. Employment status was also related significantly to PA for both sexes in the three studies in which this association was examined [20, 22, 27], although with mixed dependencies on employer type (government vs. non-governmental) [22, 27].

In the three studies in which it was examined, the relationship between women’s BMI and PA was not significant [18, 24, 29]. In one of these studies, however, physically active women had a significantly lower average BMI [18]. Data on the relationship between BMI and PA for both sexes combined, provided in four articles, were mixed [20, 27, 31, 32].

Four of the five studies examining country- or region-level differences in PA revealed significant differences for both sexes combined [18, 21, 22, 25]; the two studies in which sex-specific analyses were conducted also showed significant differences for women [18, 21]. Residence (urban vs. rural) was also considered in two of these studies, and had no significant effect on the PA of men and women combined [22, 25].

The relationship between the income level and PA was examined in five studies, and no significant relationship was found among men and women combined [19, 21, 22, 25, 31] or among women alone [22, 25].

### Health beliefs

The proportions of respondents reporting perceived barriers, perceived benefits, and/or self-efficacy were provided in four articles [19–21, 28]. These studies were conducted with Egyptian and Saudi college students [21] and patient populations [19, 20, 28]. Relationships of these beliefs to PA (among both sexes combined) were examined in two studies [20, 28]. Results regarding percentages of respondents confirming perceived barriers, perceived benefits, or self-efficacy are presented as non-significant results.

#### Perceived barriers

Five perceived barriers were investigated via questionnaire in four studies and reported on in at least two articles. They were the lack of time, lack of resources, lack of access, lack of social support, fear of criticism, and environmental barriers. Lack of time was examined in all four studies [19–21, 28], and its relationship to PA was explored and found to be non-significant in two studies [20, 28]. In two of the three studies, more than 80% of respondents reported lack of time. The lack of resources was considered in two studies [19, 28]. It both studies, it was associated positively with the modifying factor of income, but not to age or educational level. Less than half of female respondents reported this perceived barrier. Lack of social support was considered in three studies [19, 21, 28], and was not related to the modifying factors of age, educational level, income, or marital status in two of these studies [19, 28]. The fear of injury was considered in three studies [19, 21, 28]; in two of these studies [19, 28], it was reported by less than 30% of female respondents and was associated negatively with age and educational level.

#### Perceived benefits

Perceived benefits were considered in two studies [20, 21]. No significant association of PA with weight control/obesity prevention, health maintenance, or fun and enjoyment was found in either study.

#### Self-efficacy

Self-efficacy was considered in three studies [19, 21, 28]. The potential negative effects of the lack of willpower, lack of skills, and lack of energy/power on PA were reported on in more than one article.

In the two studies in which the lack of willpower was examined, 48.6% and 78.9% of female respondents, respectively, reported this factor and this variable was not associated significantly with age, marital status, or educational level [19, 28]. Sex-specific data on the lack of skills was provided in three studies [19, 21, 28], less than half of female respondents reported this factor in two of these studies [19, 28]. For both sexes together, the lack of skills was associated negatively with the educational and not with marital status. The lack of energy was not associated significantly with age, marital status, or income in any study.

Health beliefs were compared between women and men in two studies [19, 28]. In both studies, significantly more women than men reported a lack of social support and women were significantly more likely than men to report a lack of skills; no gender difference was found for the fear of injury or lack of willpower (Table 3).

#### Cues to action

Among the few cues to action considered in the studies, medical advice to engage in PA was considered in two studies and found to not be associated significantly with PA [20, 28].

## Discussion

This systematic review confirmed the low prevalence of PA among women in GCC countries, ranging from nearly 0% to about 50% [33–35]. Variation in this prevalence was due in part to differences in the subpopulations examined and PA measurement instruments used. Inconsistency in the definition and measurement of PA has been documented [33]. State-of-the-art instruments, such as the World Health Organization’s Global Physical Activity Questionnaire (GPAQ) and the International Physical Activity Questionnaire (IPAQ) [36, 37], were used to measure PA in only three of the studies included in this review. We recommend the use of a unified measure to examine PA and related factors in GCC countries to aid comparison and the monitoring of PA prevalence.

This review differs from previous reviews in terms of the population examined and because the HBM was used to identify factors influencing PA. Below, we first synthesize the findings and discuss implications for future research and policy. The findings should be interpreted with caution because the sample was small and because of variation in the populations studied and definition and measurement of PA.

The modifying factors considered in multiple studies were the age, marital status, educational level, employment status, BMI, study region, income level, health status, and residence. PA levels were lower in women aged ≤ 25 years and diminished around the age of 40–60 years, depending on the study. Employment status was related positively to PA. PA was not related to the marital status, educational level, BMI, or income. Results for the relationship of PA to health status and region were few and inconclusive, as might be expected given the nonspecific nature of these variables.

Relationships of the HBM constructs of perceived susceptibility and perceived severity to PA were not reported on in any included article. Given the low prevalence of PA among women in GCC countries and the importance of PA as a morbidity and mortality risk factor [1, 2], it is remarkable that associations of these beliefs with PA have not received research attention. Only four of the 15 included articles provided data on health beliefs, and only two articles provided data on the relationships of these beliefs to PA. As both of these studies were conducted with mixed-sex populations, we cannot present any evidence for relationships between health beliefs and PA among women in GCC countries. Given the low prevalence of PA in these countries, especially among a women, robust research on these relationships is urgently needed.

The few data on the relationships between health beliefs and PA that were examined in this review indicate that these relationships are complex, for the general population and for women specifically. For instance, the lack of time was the most commonly reported barrier, but was not related to PA in either of the two studies in which this association was examined. A significant relationship was found only for the fear of injury. Thus, the health beliefs related to PA engagement among women (and men) in GCC countries are poorly understood, and further research is urgently needed.

More women than men in the review sample reported the lack of social support and skills, and these factors may explain the gender difference in PA prevalence in this population. The fear of injury and lack of willpower did not differ according to gender. Evidence for such differences in the lack of resources, time, and energy is inconclusive. However, gender differences in health beliefs were examined in only two studies. Thus, evidence explaining the low relative prevalence of PA among women in GCC countries is very scarce, even though significant sex differences in prevalences were observed in all studies conducted with general populations. Although culture-related gender differences are commonly discussed and provide intrinsic contextual variables that could plausibly explain this gender difference in PA prevalence [4–7], we found remarkably little scientific evidence on resulting differences in health beliefs. The GCC region is distinct in terms of climate, cultural, and religious factors, and more rigorous examination of region-specific health beliefs is needed. Given the current lack of evidence, exploratory qualitative research should be performed before further surveys are conducted with instruments for the measurement of health beliefs that have been validated in other contexts.

Thus, our first policy recommendation is to commission rigorous scientific studies for the identification of health beliefs and modifying factors underlying the low PA prevalence in the general adult population and specifically among women in GCC countries. Such studies must be conducted with valid, uniform PA measurement instruments, such as the GPAQ and IPAQ [33–35]. Second, policy efforts should target young-adult and elderly women, among whom the prevalence of PA is particularly low. Likewise, specific policy measures to promote PA among unemployed women are needed; moreover, increasing the female employment rate may effectively increase the PA prevalence among women in GCC countries. Although the relationships between health beliefs and PA are poorly understood, our findings suggest that the promotion of social support and skill acquisition for these women’s engagement in PA could effectively reduce the gender gap in the PA prevalence.

## Limitations

This review is limited by the small sample and the variability in PA measurement instruments and research methods used. The study populations also varied, despite the linguistic and religious homogeneity of the study region, which compromised the generation and validity of GCC-wide evidence. In search of robust scientific evidence, we included only English-language peer-reviewed scientific publications in this review; we may have missed data published in Arabic and/or in the gray literature.

## Conclusions

Robust qualitative and quantitative research on PA among women and general populations in the GCC countries, conducted with standardized and validated measurement instruments such as the GPAQ, is urgently needed. Definitive evidence for relationships of many obvious factors and health beliefs to PA in this population is lacking. This review revealed that unemployed, young-adult, and elderly women are less likely to be physically active, and women are more likely than men to believe that they lack social support and skills for PA engagement.

## Supplementary Information

**Additional file 1. **

**Additional file 2:** **Appendix 2.** Search Query per database.

**Additional file 3**.

**Additional file 4**.

## Data Availability

The datasets used and/or analyzed during the current study are available from the Medline (Ovid), EMBASE, Cochrane Central, Web of Science, and Google Scholar databases.

## Abbreviations

PA: Physical activity
GCC: Gulf Cooperation Council
HBM: Health belief model
MMAT: Mixed Methods Appraisal Tool
BMI: Body mass index
GPAQ: Global Physical Activity Questionnaire
IPAQ: International Physical Activity Questionnaire
