# Supplementary material for: A theory-informed systematic review to understand physical activity among women in Gulf Cooperation Council countries
Source: BMC Public Health. 2023 May 30;23:1009. doi: 10.1186/s12889-023-15725-5 (PMC10227989; doi:10.1186/s12889-023-15725-5)
Supplement: Supplementary file 1 — Additional file 1. [file 12889_2023_15725_MOESM1_ESM.docx]

| **Section and Topic** | **Item #** | **Checklist item** | **Location where item is reported** |
| --- | --- | --- | --- |
| **TITLE** | | |  |
| Title | 1 | A THEORY-INFORMED SYSTEMATIC REVIEW TO UNDERSTAND  PHYSICAL ACTIVITY AMONG WOMEN IN GCC COUNTRIES | 1 |
| **ABSTRACT** | | |  |
| Abstract | 2 | See abstract of revised manuscript | 2,3 |
| **INTRODUCTION** | | |  |
| Rationale | 3 | to investigate PA and factors associated with PA for the female population in GCC countries. | 5 |
| Objectives | 4 | systematically identify the associations between constructs of the HBM and PA among women in GCC  countries, as reported in empirical scientific literature. | 5 |
| **METHODS** | | |  |
| Eligibility criteria | 5 | Studies were eligible for inclusion if they (1) assessed the association between physical activity  as a dependent variable and one or several factors that influence physical activity as  independent variables; (2) conducted within the Gulf States countries,  namely Saudi Arabia, Bahrain, Kuwait, Oman, Qatar, United Arab of Emirates; (3)  The target population was adults including women (but not necessarily restricted to  women only) and (4) published in English.  Studies were excluded if (1) reporting on the prevalence of physical (in) activity only  without corresponding factors; (2) the included population was restricted to a specific  disease (as opposed to the (female) population in general); (3) there was no explicit  reporting on adults (but for instance on children and adolescents), or; (4)  physical activity was considered as an independent variable. Grey literature was also excluded. | 6,7 |
| Information sources | 6 | MEDLINE (Ovid), EMBASE, the Cochrane CENTRAL, Web of science, and the Google Scholar | 6 |
| Search strategy | 7 | See Appendix 2 |  |
| Selection process | 8 | Two researchers (LAO and SAA) independently assessed the titles and abstracts of the remaining  articles (n=1421) for eligibility using the aforementioned inclusion and exclusion criteria (Figure 2).  A total of 112 articles remained included after this step. The next round of full-text reading led to  the exclusion of 90 articles, after which 22 remained included. In case of disagreement or  uncertainty (n=7), the third and the fourth authors were consulted (JVK and JMC). This led to a final set  of 15 included articles. (Figure 2) | 6,7 |
| Data collection process | 9 | See above. | 7 |
| Data items | 10a | Physical Activity was the outcome of interest. All measures accepted as long as physical activitiy was the  Dependent variable. | 7 |
|  | 10b | Author and year, Study Design, Instrument (if any, e.g. a questionnaire or a step counter),  Physical activity, Country, Study population, Modifying factors, Perceived susceptibility,  Perceived severity, Perceived benefits, Perceived barriers, Self-efficacy, Cause to action,  and taking action, all for the general study population, for women only and for women vs men) | 7 |
| Study risk of bias assessment | 11 | The quality of the studies was assessed using the MMAT tool. Biases in study population (e.g. only college  students or primary care patients included) were explicitly noted and reported. The nature of the research  question and reporting does not require further quantitative bias correction  (there is no quantitative meta analysis). | 7 |
| Effect measures | 12 | All outcome measures were accepted (see above and Appendix 4), for completeness of the review. | 7 |
| Synthesis methods | 13a | All results regarding modifying factors and health beliefs for the general population, women only, and  women versus men were included, significant or not, for completeness of the systematic review. | 7 |
|  | 13b | We didnt process any of the data beyond retrieval from the included sources. | 7 |
|  | 13c | The data were tabulated in MS Excel, one row per study, which included all data retrieved and comments. | 7 |
|  | 13d | We only presented synthesis for factors and associations reported in more than one study.  Results were only considered inclusive (for a certain population) if all (non) significant and with the same  sign. | 9 |
|  | 13e | We didnt explore causes of heterogeneity among study results but address it in the discussion |  |
|  | 13f | No sensitivity analyses conducted. |  |
| Reporting bias assessment | 14 | For reasons of completeness and inclusiveness, and because of the lower number of studies,  we included all data and call for caution in the discussion. |  |
| Certainty assessment | 15 | No further methods implemented. |  |
| **RESULTS** | | |  |
| Study selection | 16a | See Figure 2 of the revised manuscript. | Figure 2 |
|  | 16b | Related literature is introduced in the introduction. The full data extracion files are available upon request. | 3,4 |
| Study characteristics | 17 | See Table 1 and the references of the revised manuscript. | Table 1 |
| Risk of bias in studies | 18 | See Appendix 3 for MMAT scores and above for further comments related to bias. |  |
| Results of individual studies | 19 | See Table 1 of the revised manuscript. | Table 1 |
| Results of syntheses | 20a | See the results section of the revised manuscript and the discussion for interpration and caution. | 12,13,14 |
|  | 20b | The results section systematically reviews evidence for all modifying factors, and health beliefs,  In relation to physical activity, based on the presentation of evidence in Tables 2 and 3. | 12,13,14 |
|  | 20c | Bias assessment is not part of the results as the nature and number of studies didnt allow use  of commonly accepted methods. Possible biases, e.g. for specific study populations, are  covered in the discussion. |  |
|  | 20d | No sensitivity analyses conducted . |  |
| Reporting biases | 21 | Risk of bias due to missing results was not assessed. |  |
| Certainty of evidence | 22 | The robustness of the presented evidence is explicitly and transparently addressed in the discusion | 14,15 |
| **DISCUSSION** | | |  |
| Discussion | 23a | See discussion section of the revised manuscript | 14-17 |
|  | 23b | See limitations section of revised manuscript | 18 |
|  | 23c | See limitations section of revised manuscript | 18 |
|  | 23d | See discussion and conclusion sections of revised manuscript | 14-18 |
| **OTHER INFORMATION** | | |  |
| Registration and protocol | 24a | The review was not registered. |  |
|  | 24b | The review protocol is included in the methods setion of the revised manuscript and the appendices  references therein. | 6,7 |
|  | 24c | N.A. |  |
| Support | 25 | No financial support was obtained. |  |
| Competing interests | 26 | The review authors have no competing interests. |  |
| Availability of data, code and other materials | 27 | The data collection instrument and data included are available in Appendix 4. |  |

*From:*  Page MJ, McKenzie JE, Bossuyt PM, Boutron I, Hoffmann TC, Mulrow CD, et al. The PRISMA 2020 statement: an updated guideline for reporting systematic reviews. BMJ 2021;372:n71. doi: 10.1136/bmj.n71

For more information, visit: <http://www.prisma-statement.org/>
