# Supplementary material for: A theory-informed systematic review to understand physical activity among women in Gulf Cooperation Council countries
Source: BMC Public Health. 2023 May 30;23:1009. doi: 10.1186/s12889-023-15725-5 (PMC10227989; doi:10.1186/s12889-023-15725-5)
Supplement: Supplementary file 2 — Additional file 2: Appendix 2. Search Query per database. [file 12889_2023_15725_MOESM2_ESM.docx]

Appendix 2: Search Query per database

| *Databases* | *References* | *After de-duplication* |
| --- | --- | --- |
| Embase.com | 1746 | 1704 |
| Medline (Ovid) | 1153 | 287 |
| Web of Science | 2223 | 1401 |
| Cochrane CENTRAL | 72 | 20 |
| Google Scholar | 200 | 119 |
| **Total** | **5394** | **3531** |

**Embase.com**

('physical activity'/exp OR 'physical inactivity'/exp OR 'sedentary lifestyle'/de OR 'television viewing'/de OR 'sport'/exp OR 'exercise'/exp OR ((physical* NEAR/3 (activ* OR inactiv*)) OR sport* OR exercise* OR fitness* OR climbing* OR cycling* OR fighting* OR jogging* OR jumping* OR lifting-effort* OR running* OR stretching* OR swimming* OR walking* OR ((weight) NEAR/3 (bearing* OR lifting*)) OR athletic* OR baseball* OR basketball* OR bowling* OR diving* OR football* OR golf* OR hockey* OR horseback-riding* OR jogging* OR mountaineering* OR rowing* OR rugby* OR running* OR skateboarding* OR skating* OR skiing* OR soccer* OR triathlon* OR volleyball* OR yoga* OR body-building* OR ((circuit OR endurance OR interval OR resistance) NEAR/3 (training*)) OR pilates* OR plyometric OR gymnastic* OR bicycl* OR ((riding OR ride*) NEAR/3 (bike*)) OR boxing* OR martial-art* OR tennis* OR wrestling* OR cricket*):ab,ti) AND ('Persian Gulf'/exp OR (Bahrain* OR Iraq* OR Kuwait* OR Oman* OR Qatar* OR Saudi* OR United-Arab-Emirates* OR Abu-Dhabi* OR Ajman* OR Dubai* OR Sharjah* OR gulf-state* OR Persian-gulf*):ab,ti) NOT (('male'/exp OR (male* OR man OR men OR boy*):ab,ti) NOT ('female'/exp OR (female* OR women OR woman OR girl*):ab,ti))

**Medline Ovid**

(exp Sports/ OR exp Exercise/ OR Sedentary Lifestyle/ OR ((physical* ADJ3 (activ* OR inactiv*)) OR sport* OR exercise* OR fitness* OR climbing* OR cycling* OR fighting* OR jogging* OR jumping* OR lifting-effort* OR running* OR stretching* OR swimming* OR walking* OR ((weight) ADJ3 (bearing* OR lifting*)) OR athletic* OR baseball* OR basketball* OR bowling* OR diving* OR football* OR golf* OR hockey* OR horseback-riding* OR mountaineering* OR rowing* OR rugby* OR running* OR skateboarding* OR skating* OR skiing* OR soccer* OR triathlon* OR volleyball* OR yoga* OR body-building* OR ((circuit OR endurance OR interval OR resistance) ADJ3 (training*)) OR pilates* OR plyometric OR gymnastic* OR bicycl* OR ((riding OR ride*) ADJ3 (bike*)) OR boxing* OR martial-art* OR tennis* OR wrestling* OR cricket*).ab,ti.) AND (Bahrain/ OR Iraq/ OR Kuwait/ OR Oman/ OR Qatar/ OR Saudi Arabia/ OR United Arab Emirates/ OR (Bahrain* OR Iraq* OR Kuwait* OR Oman* OR Qatar* OR Saudi* OR United-Arab-Emirates* OR Abu-Dhabi* OR Ajman* OR Dubai* OR Sharjah* OR gulf-state* OR Persian-gulf*).ab,ti.) NOT ((Male/ OR Men/ OR (male* OR man OR men OR boy*).ab,ti.) NOT (Female/ OR Women/ OR (female* OR women OR woman OR girl*).ab,ti.))

**Web of Science**

TS=((((physical* NEAR/2 (activ* OR inactiv*)) OR sport* OR exercise* OR fitness* OR climbing* OR cycling* OR fighting* OR jogging* OR jumping* OR lifting-effort* OR running* OR stretching* OR swimming* OR walking* OR ((weight) NEAR/2 (bearing* OR lifting*)) OR athletic* OR baseball* OR basketball* OR bowling* OR diving* OR football* OR golf* OR hockey* OR horseback-riding* OR mountaineering* OR rowing* OR rugby* OR running* OR skateboarding* OR skating* OR skiing* OR soccer* OR triathlon* OR volleyball* OR yoga* OR body-building* OR ((circuit OR endurance OR interval OR resistance) NEAR/2 (training*)) OR pilates* OR plyometric OR gymnastic* OR bicycl* OR ((riding OR ride*) NEAR/2 (bike*)) OR boxing* OR martial-art* OR tennis* OR wrestling* OR cricket*)) AND ((Bahrain* OR Iraq* OR Kuwait* OR Oman* OR Qatar* OR Saudi* OR United-Arab-Emirates* OR Abu-Dhabi* OR Ajman* OR Dubai* OR Sharjah* OR gulf-state* OR Persian-gulf*)) NOT (((male* OR man OR men OR boy*)) NOT ((female* OR women OR woman OR girl*))))

**Cochrane CENTRAL**

(((physical* NEAR/3 (activ* OR inactiv*)) OR sport* OR exercise* OR fitness* OR climbing* OR cycling* OR fighting* OR jogging* OR jumping* OR lifting-effort* OR running* OR stretching* OR swimming* OR walking* OR ((weight) NEAR/3 (bearing* OR lifting*)) OR athletic* OR baseball* OR basketball* OR bowling* OR diving* OR football* OR golf* OR hockey* OR horseback-riding* OR mountaineering* OR rowing* OR rugby* OR running* OR skateboarding* OR skating* OR skiing* OR soccer* OR triathlon* OR volleyball* OR yoga* OR body-building* OR ((circuit OR endurance OR interval OR resistance) NEAR/3 (training*)) OR pilates* OR plyometric OR gymnastic* OR bicycl* OR ((riding OR ride*) NEAR/3 (bike*)) OR boxing* OR martial-art* OR tennis* OR wrestling* OR cricket*):ab,ti) AND ((Bahrain* OR Iraq* OR Kuwait* OR Oman* OR Qatar* OR Saudi* OR United-Arab-Emirates* OR Abu-Dhabi* OR Ajman* OR Dubai* OR Sharjah* OR gulf-state* OR Persian-gulf*):ab,ti) NOT (((male* OR man OR men OR boy*):ab,ti) NOT ((female* OR women OR woman OR girl*):ab,ti))

**Google Scholar**

" ( physicalactivity OR inactivity OR sport OR sports OR exercise OR fitness OR jogging OR running OR walking OR basketball OR football OR hockey OR rugby OR soccer OR yoga OR wrestling OR cricket) AND ( Bahrain OR Iraq OR Kuwait OR Oman OR Qatar OR Saudi OR "United Arab Emirates" OR "gulf|Persian state") AND ( female OR females OR women OR girls)
